# Immethridine, histamine H_3_-receptor (H_3_R) agonist, alleviated experimental autoimmune encephalomyelitis via inhibiting the function of dendritic cells

**DOI:** 10.18632/oncotarget.20500

**Published:** 2017-08-24

**Authors:** Yaru Shi, Zhenlong Li, Ran Chen, Jiang Zhang, Xuefei Hu, Cong He, Qiong Su, Hongdou Ma, Hua Ren, Min Qian, Shufang Cui, Wenzheng Jiang

**Affiliations:** ^1^ Shanghai Key Laboratory of Regulatory Biology, School of Life Sciences, East China Normal University, Shanghai, China; ^2^ Laboratory Animal Center, Second Military Medical University, Shanghai, China

**Keywords:** multiple sclerosis, EAE, H3R, immethridine, dendritic cell

## Abstract

Multiple sclerosis (MS) is an inflammatory disease that is characterized by immune-mediated demyelination and degeneration of the central nervous system (CNS). Experimental autoimmune encephalomyelitis (EAE) is the preferential experimental rodent model for MS. Previous study demonstrated histamine H3 receptor (H3R) was an important factor in pathophysiology of EAE and immethridine was the most selective agonist of H3R. However, whether immethridine has therapeutic effect on EAE and its mechanism remained to be defined. Here we constructed EAE mouse model by immunization of MOG_35-55_ peptides with complete Freund’s adjuvant, immethridine was used to treat EAE and its therapeutic effect was evaluated. The results showed that the treatment of immethridine could alleviate EAE. The percentage of Th1 and Th17 in the spleen from the treated EAE mice decreased and the surface molecules such as CD40, CD86 or MHCII on dendritic cells (DCs) were also down-regulated. To understand the effect of immethridine on DCs, bone marrow-derived DCs were prepared and the immunological functions were analyzed. The data demonstrated that immethridine could change the expression profiles of cytokines in DCs and inhibit the expression of the co-stimulatory molecules such as CD40 and CD86. Furthermore, immethridine also inhibited the antigen-presenting function of DCs and T cell differentiation induced by DCs. Signaling pathway analysis demonstrated that the phosphorylation of NF-κB p65 but not ERK1/2 in DCs was inhibited after the treatment of immethridine. These data strongly suggested that immethridine could inhibit the function of DCs and indicated the therapeutic potential on EAE.

## INTRODUCTION

Multiple sclerosis (MS) belongs to a T cell-mediated autoimmune disease which is characterized by chronic inflammatory demyelination and neurodegeneration of the central nervous system (CNS) [1]. The young adults between 20 and 40 years of age are usually onset [2]. The socioeconomic importance of the disease is second only to trauma in young adults although it is usually not life-threatening [3-5]. Due to disease complexity and heterogeneity, the pathogenesis of MS remains unknown and the specific effective treatments have not yet been developed. Experimental autoimmune encephalomyelitis (EAE), which shares many pathological and histological similarities with MS and is a widely used animal model for MS, provides crucial evidence of autoimmune mechanisms [6-8].

Dendritic cells (DCs) are the most potent antigen presenting cells (APCs) in both the innate and adaptive immune responses. In peripheral tissues, immature DCs have strong ability to capture antigens (Ags) and undergo a maturation process upon contact with stress factors such as TNF-α or lipopolysaccharide (LPS). The matured DCs lose their capacity to process Ags, but they have higher level of co-stimulatory molecule expression, produce cytokines, and migrate to the lymphoid organs to prime naive Ag-specific T cells [9, 10]. In EAE, DCs play a dual role. Firstly, they activate naïve antigen-specific CD4^+^ T cells and induce them to differentiate into Th1 and Th17 effector cells in peripheral lymphoid organs. Secondly, DCs re-stimulate migrating encephalitogenic T cells in the perivascular space of the CNS and facilitate their infiltration into the CNS parenchyma [11].

Histamine [2-(4-imidazole) ethylamine] is a ubiquitous inflammatory mediator and has diverse physiological processes, such as neurotransmission, secretion of pituitary hormones, and regulation of the gastrointestinal and circulatory systems, etc. It has been reported that histamine can modulate immune responses and plays a key regulatory role in EAE or MS [12]. Histamine exerts several physiological functions by activating distinct subtypes of histamine receptors. The histamine receptors, such as H1, H2, H3, H4 receptors, belong to members of G protein-coupled receptor (GPCR) families [13, 14]. H3R is one of the important histamine receptors and it was discovered as the autoreceptor, which regulated the secretion and generation of histamine [15]. The expression of H3R is expressed not only in CNS, but also in other tissues and cells such as lung, cardiovascular system, intestine or DCs [12]. Teuscher et al. have reported that H3R-KO mice developed more severe EAE disease compared with wide-type B6 mice, [16]. H3R antagonists have been applied for the therapy on several diseases and CNS disorders, such as attention-deficit hyperactivity disorder (ADHD), Alzheimer’s disease, epilepsy, schizophrenia, and obesity. On the other hand, the therapeutic applications of H3R agonists for myocardial ischemia, inflammatory, and gastric acid related diseases have also been reported [17]. Immethridine dihydrobromide is a strong and highly selective histamine H3 receptor agonist that displays 300-fold selectivity over the H4 receptor and does not bind to H1 or H2 receptors at concentrations up to 10 μM [18], immethridine plays an opposite effect against the gastric lesions induced by HCL in rats [19, 20].

So far, the pathogenesis of MS or EAE remains unknown, the effective treatments have not yet been developed. Since H3R-KO mice have developed more serious EAE [16], it is very meaningful to address whether immethridine, a strong and highly selective agonist of H3R, has the therapeutic effect on EAE. In this study, the data showed that immethridine inhibited the functions of DC cells and reduced the severity of EAE.

## RESULTS

### The treatment of immethridine reduced the severity of EAE

**Figure 1:**
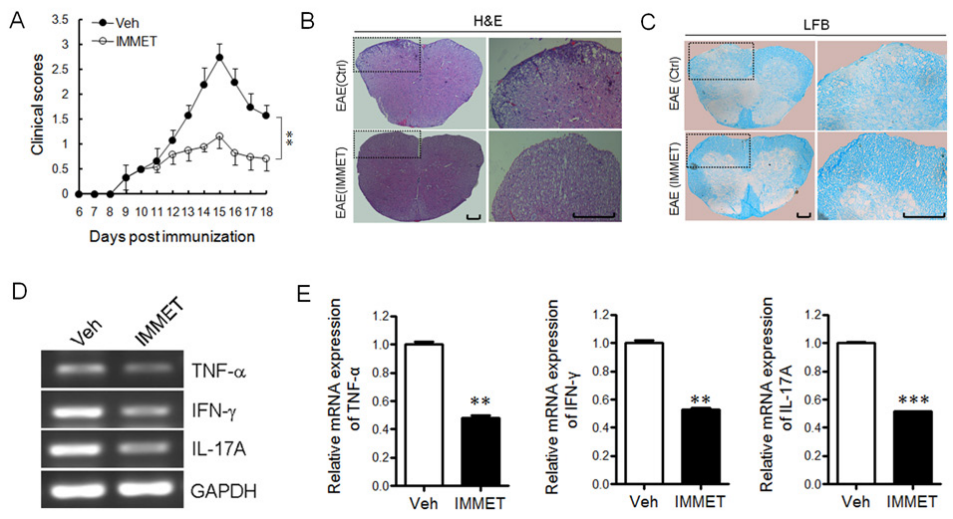
H3R agonist immethridine could alleviate the severity of EAE **(A)** EAE mice were treated with immethridine (15 mg/kg) every other day via i.p. from day 8 post-immunization. Control groups were given PBS injection. Data are mean±SD (n=6). **p<0.01 versus vehicle. **(B)** H&E staining and **(C)** LFB staining of paraffin sections of spinal cords. After the mice (n=6) were sacrificed on day 21, the spinal cord samples were isolated from vehicle and immethridine–treated EAE mice and fixed in 4% (w/v) paraformaldehyde for 48h, then the paraffin-embedded sections were stained with hematoxylin-eosin or luxol fast blue. Scale bar, 100 μm. **(D)** RT-PCR analysis of the pro-inflammatory cytokines of splenocytes. The splenocytes from vehicle and immethridine–treated EAE mice were collected after the mice were sacrificed on day 21 and the total RNAs were extracted. RT-PCR was performed with the specific primers of cytokines and the PCR products were analyzed on a 1% agarose gel stained with ethidium bromide. **(E)** Q-PCR analysis of the pro-inflammatory cytokines of splenocytes. **p<0.01, ***p<0.001 versus vehicle. Data are representative of three independent experiments.

To evaluate whether H3R agonist immethridine could alleviate the severity of EAE, EAE mice were induced with MOG_35-55_ immunization and the treatment of immethridine was performed. The EAE mice treated with immethridine had lower clinical scores than the control EAE mice (Figure 1A), which showed that the treatment of immethridine significantly reduced the severity of EAE. Histological examination of the spinal cords was performed at day 21. Compared to the control EAE mice, the treated EAE mice with immethridine had fewer inflammatory infiltrates in spinal cord (Figure 1B) and a decrease of demyelination in histological analysis of spinal cord (Figure 1C). Splenocytes were isolated from immethridine-treated and untreated EAE mice and the expression of pro-inflammatory cytokines was analyzed by RT-PCR (Figure 1D) and Q-PCR (Figure 1E), respectively. The result showed that lower level of TNF-α, IFN-γ and IL-17A was detected in immethridine-treated mice. Taken together, these data suggested that immethridine could alleviate the severity of EAE.

### Immethridine treatment affected the immune cells in EAE mice

**Figure 2:**
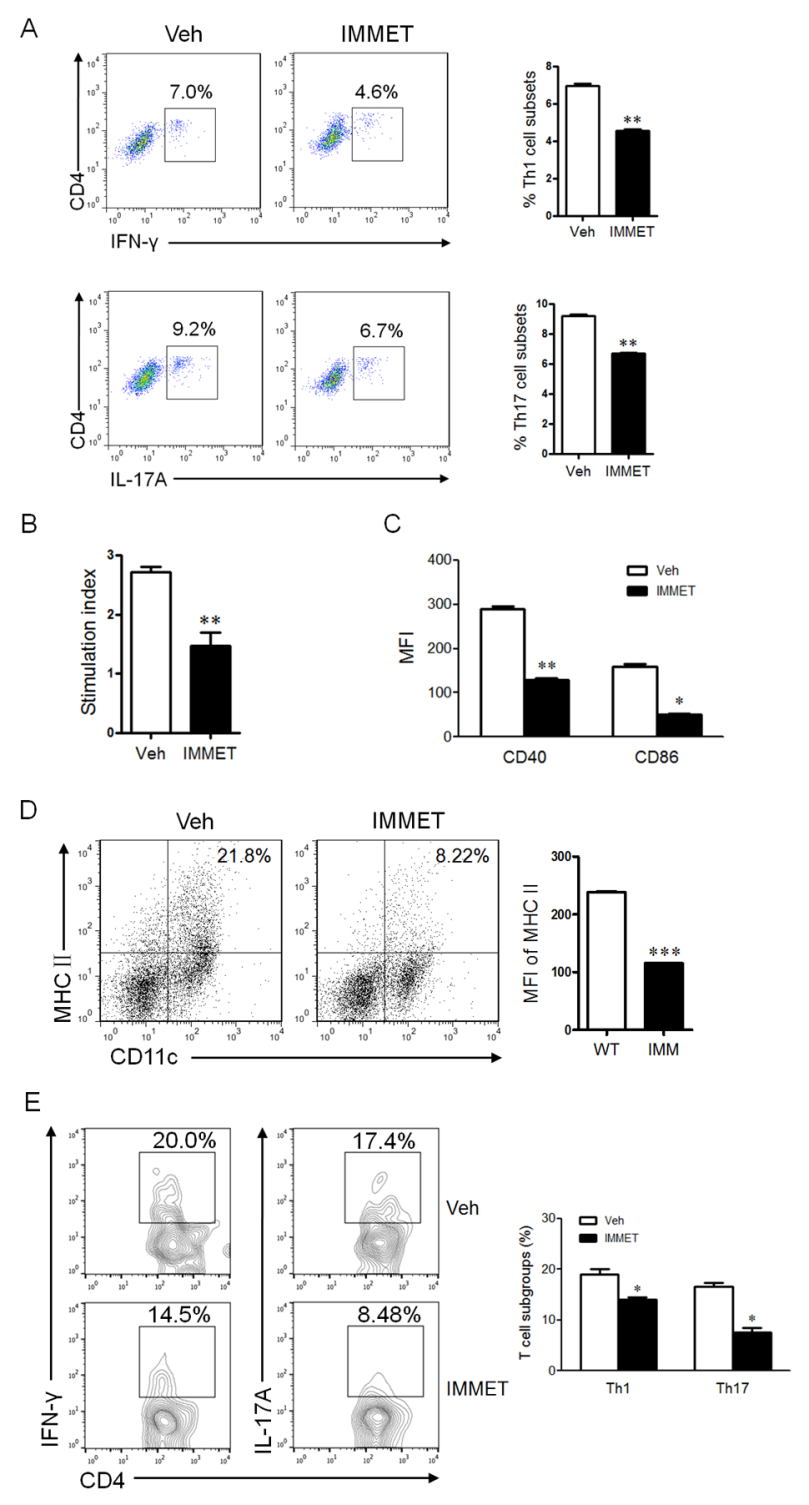
The treatment of immethridine could affect the differentiation and function of immune cells in EAE mice **(A)** FACS analysis of CD4^+^ T cell subsets in splenocytes of EAE mice (n=6). Splenocytes were prepared and Th1 and Th17 cells were analyzed by intracellular staining of IFN-γ and IL-17A, respectively, in the CD4^+^ gate. *p<0.05, **p<0.01 versus vehicle. **(B)** MTT analysis of MOG_35-55_-specific T cell proliferation. Splenocytes of EAE mice (n=6) were isolated and cultured at the present or absent of MOG_35-55_ peptides for 72h and T cell proliferation was measured using a MTT Cell Proliferation Assay Kit. The data were shown at stimulation index. **p<0.01 versus vehicle. **(C)** FACS analysis of expression of co-stimulatory molecules on DCs in splenocytes of EAE mice (n=6). Splenocytes were prepared and stained with fluorescent labeled-antibodies against CD11c, CD40 or CD86, the stained cells were analyzed on flow cytometry. *p<0.05, **p<0.01 versus vehicle. **(D)** FACS analysis of expression of MHCII on DCs in spinal cord of EAE mice (n=6). Immune cells in spinal cord were prepared and stained with fluorescent labeled-antibodies against CD11c, MHCII, the stained cells were analyzed on flow cytometry. ***p<0.001 versus vehicle. **(E)** FACS analysis of CD4^+^ T cell subsets in spinal cord of EAE mice (n=6). The lymphocytes from spinal cord were prepared and Th1 and Th17 cells were analyzed by intracellular staining of IFN-γ and IL-17A, respectively, in the CD4^+^ gate. *p<0.05 versus vehicle. Data are representative of three independent experiments.

Th1 and Th17 cells play a crucial pathological role in EAE [21, 22]. The splenocytes were isolated from spleen of immethridine-treated and control EAE mice on day 21 post-immunization and analyzed for Th1, Th17 and Treg by intracellular staining of IFN-γ, IL-17A or Foxp3, respectively. We found that the percentage of Th1 and Th17 subgroups in CD4^+^ population was significantly lower in the spleen of immethridine-treated mice compared to control EAE mice (Figure 2A), but there was no significant difference for Treg cells (data not shown). Moreover, splenocytes were isolated from EAE mice and MOG_35-55_-specific T cell proliferation was also investigated, the data showed that a lower level of cell proliferation was detected for T cells from immethridine-treated mice (Figure 2B). DCs play an important role in the development of inflammatory responses in MS/EAE [11], we therefore analyzed the expression of co-stimulatory molecules on DCs in splenocytes from immethridine-treated and control EAE mice. Interestingly, the lower expression of co-stimulatory molecules such as CD40 and CD86 on DCs from immethridine-treated EAE mice was detected (Figure 2C). The expression level of MHCII on DCs in spinal cord was also detected and the expression of MHCII on DCs in spinal cord from immethridine-treated EAE mice was down-regulated compared to control EAE mice (Figure 2D), which indicated that immethridine could affect the function of DCs. Furthermore, the immune cells and CD4^+^ T cell subsets in spinal cord were also analyzed by FACS. The data showed that there was no significant difference for the numbers of DCs, CD4 and CD8^+^ T cells between immethridine-treated EAE mice and untreated mice (data not shown). However, there were fewer Th1 and Th17 cells in spinal cord from immethridine-treated EAE mice (Figure 2E).

### Immethridine affected the cytokine expression profiles in BMDCs

**Figure 3:**
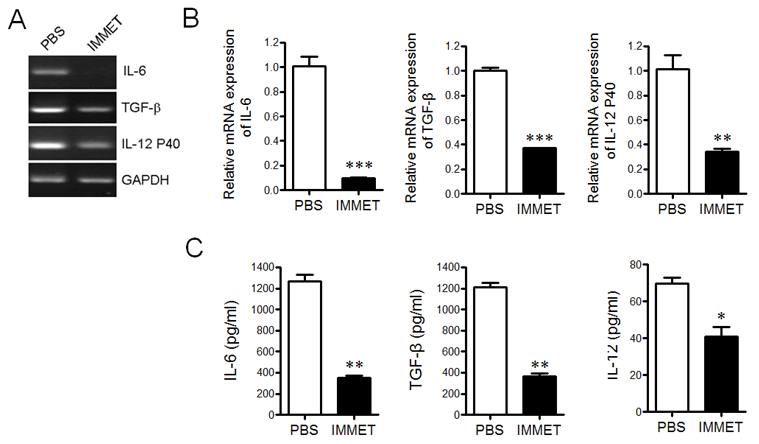
Immethridine could inhibit the cytokine secretion in BMDCs BMDCs were prepared and stimulated with immethridine (1 μM) and LPS (100 ng/ml) in 24-well plates for 6h. The total mRNAs were extracted and the transcription level of the cytokines was detected by RT-PCR **(A)** and Q-PCR **(B)**, respectively. The culture supernatant was collected and the protein level of the cytokines was measured by ELISA **(C)**. *p<0.05, **p<0.01, ***p<0.001 versus PBS. Data are representative of three independent experiments.

DCs can produce an ample repertoire of cytokines and mediate diverse immune responses through the secreted cytokines. Previously studies showed that histamine could inhibit LPS-induced IL-12 production by DCs and the effect was mediated through both H1R and H2R [23]. Although the expression of H3R on DCs was controversial, it has been detected in DCs by RT-PCR in current study and the expression level of H3R was down-regulated after DCs were activated by LPS stimulation (data not shown), which was consistent with the previous report [24]. To understand if immethridine, the highly selective agonist of H3R, could affect the cytokine expression in DCs, the bone marrow-derived dendritic cells (BMDC) were prepared. At day 6, DCs were stimulated with immethridine (1 μM) and LPS (100 ng/ml) in 24-well plates for 6h. After the cells were collected, total mRNAs were extracted and RT-PCR was performed to detect the expression of cytokines such as IL-6, TGF-β and IL-12 P40. The data showed that immethridine-treated DCs had lower expression level of IL-6, TGF-β and IL-12 P40 compared to PBS (Figure 3A), and the data were confirmed by Q-PCR (Figure 3B) and ELISA (Figure 3C). Taken altogether, these results suggested that immethridine could inhibit cytokine production in DCs.

### Immethridine inhibited DC-induced Th1/Th17 differentiation

**Figure 4:**
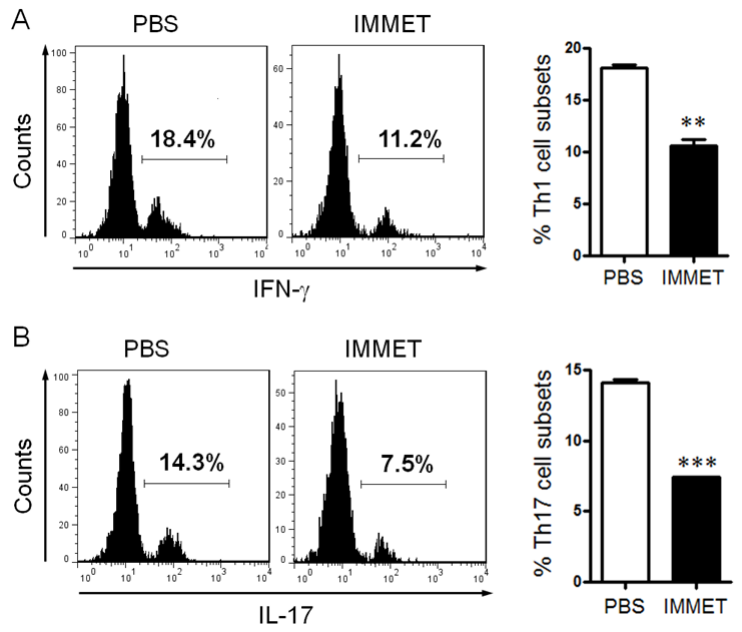
Immethridine could affect DC-induced CD4^+^ T cell differentiation BMDCs were prepared and stimulated with immethridine (1 μM) and LPS (100 ng/ml) for 24h, and naïve CD4^+^T cells were separated from splenocytes with Mouse CD4 Negative Selection Kit. After BMDCs and naïve CD4^+^T cells were co-cultured at a ratio of for 72h, Th1 cells **(A)** and Th17 cells **(B)** were analyzed by intracellular staining of IFN-γ and IL-17A in the CD4^+^ gate. **p<0.01, ***p<0.001 versus PBS. Data are representative of three independent experiments.

DCs play a vital role in initiating the innate immune response by production of cytokines, these cytokines play an important role in promoting CD4^+^ T cells to differentiate into different T cell subsets such as Th1, Th2, Th17, etc. To verify if the treatment of immethridine affected T cell differentiation induced by DCs, the purified naïve CD4^+^ T cells were mixed with DCs at a ratio of 10:1 for 72h. The percentage of Th1 and Th17 cells was analyzed by intracellular staining with FITC-labeled anti-IFN-γ or FITC-labeled anti-IL-17A, the data indicated that the percentage of Th1 and Th17 in CD4^+^ population was significantly lower in immethridine-treated DCs compared to the control DCs (Figure 4A and Figure 4B).

### Immethridine inhibits the expression of surface molecules and antigen-presentation ability of BMDCs

**Figure 5:**
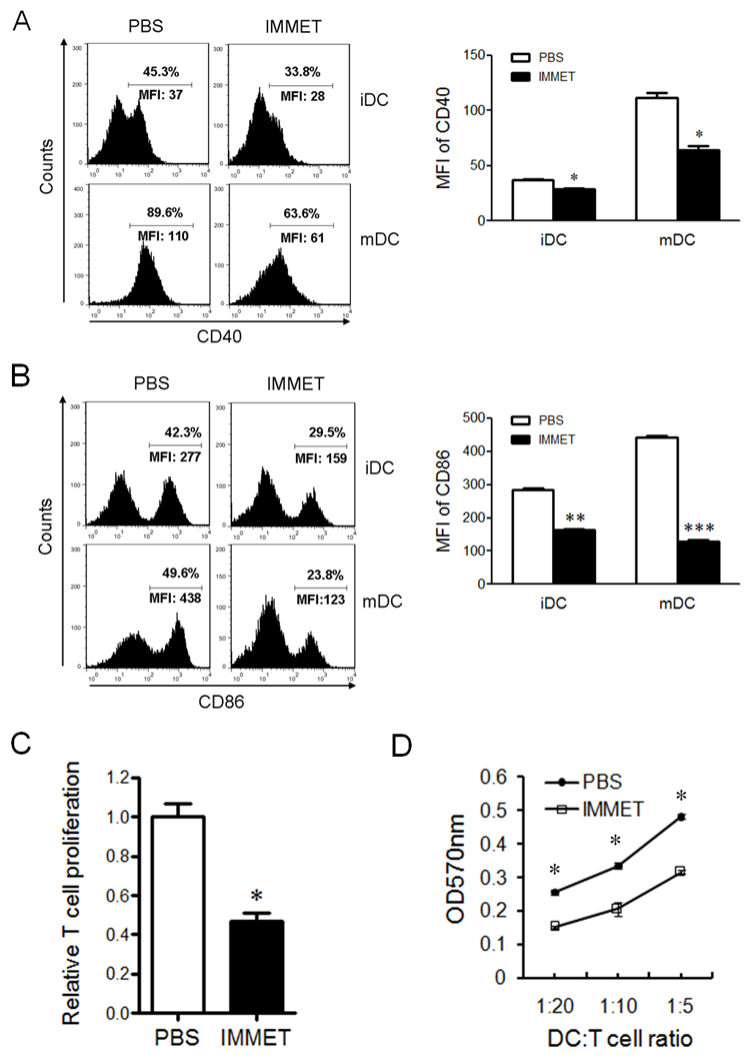
Immethridine could inhibit the function of BMDCs FACS analysis of CD40 and CD86 on BMDCs. Immature DCs were exposed to combinations of immethridine and/or LPS for 12 h, and cells were stained with a FITC-labeled anti-CD11c and PE-labeled anti-CD40 **(A)** or anti-CD86 **(B)**, then the results were analyzed by FACS. **p<0.01, ***p<0.001 versus PBS. Data are representative of three experiments. **(C)** Immethridine inhibited the capacity of antigen-presentation of DCs. MOG-pulsed DCs was co-cultured with MOG-specific CD4^+^T for 72h and T cell proliferation was detected by MTT Cell Proliferation Assay Kit. The data were shown in relative T cell proliferation. *p<0.05 versus PBS. **(D)** MLR assay. Purified T cells from BALB/C mice and immethridine-treated or untreated DCs were co-cultured for 5 days and T cell proliferation was detected by MTT Cell Proliferation Assay Kit. The data was shown in OD value at 570nm. *p<0.05 versus PBS. Data are representative of three experiments.

During the maturation process, DCs up-regulate the expression of cell surface molecules, such as CD40, CD80, and CD86, that is important for interaction with B and T cells and subsequent development of the adaptive immune response [25, 26]. The expression of surface molecules on DCs was analyzed by FACS, the date showed that immethridine could inhibit the expression of CD40 (Figure 5A), CD86 (Figure 5B) in immature or matured DCs, which indicated that the treatment of immethridine could inhibit the maturation of DCs. DCs are crucial antigen-presenting cells (APCs) for primary T-cell response, these cells are critically important in triggering an effective adaptive immune response. To understand whether immethridine affected the antigen presenting ability of DCs, MOG-pulsed DCs were co-cultured with MOG-specific CD4^+^T and T cell proliferation was detected by MTT kit (Figure 5C). The results showed that immethridine-treated DCs could induce less T cell proliferation compared to the control DCs, which indicated that the antigen-presenting ability of DCs could be inhibited by immethridine. Mixed lymphocyte reaction (MLR) was also performed and the result demonstrated that immethridine-treated DCs had lower ability to stimulate allogeneic T-cell proliferation compared to the untreated DCs (Figure 5D). Taken together, these results strongly suggested that immethridine could inhibit the immunological functions of DC cells.

### Immethridine inhibited the expression of TLR4/CD14 and phosphorylation of NF-κB p65

**Figure 6:**
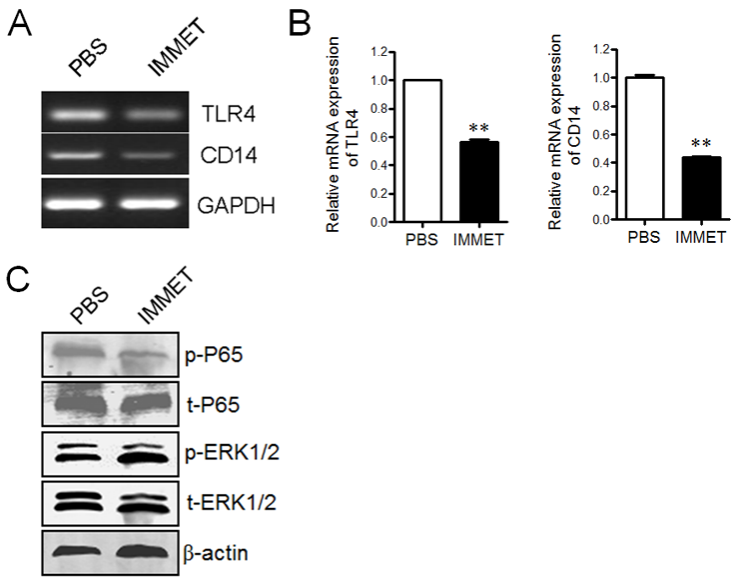
Immethridine could inhibit the signaling pathway by LPS stimulation **(A)** RT-PCR analysis of the expression of TLR4 and CD14. DCs were treated with 100 ng/ml of LPS and 1 μM of immethridine for 12h, then the total RNAs were extracted and RT-PCR was performed using the specific primers. The PCR products were analyzed on a 1% agarose gel stained with ethidium bromide. **(B)** Q-PCR analysis of the expression of TLR4 and CD14. **p<0.01 versus PBS. **(C)** Western blot analysis of signaling pathway. The cell protein was prepared after DCs were treated with immethridine, SDS-PAGE were performed and the separated protein was electro-transferred onto nitrocellulose membrane and stained by anti-mouse primary antibodies and HRP-conjugated secondary antibodies, respectively. Data are representative of three experiments.

TLR4 and co-receptor CD14 play important roles in LPS signaling pathway and involve in the expression of surface molecules on DCs [27]. The expression of TLR4 and CD14 in DCs was analyzed by RT-PCR (Figure 6A) and Q-PCR (Figure 6B), respectively. The result showed that immethridine could down-regulate the expression of TLR4 and CD14. To further understand the signaling pathway involved in the down-regulation of the surface molecules, the phosphorylation level of NF-κB p65 and ERK1/2 was analyzed by western blot, the data demonstrated that the phosphorylation of NF-κB p65 but not ERK1/2 was significantly inhibited by the treatment of immethridine (Figure 6C).

## DISCUSSION

Multiple sclerosis (MS) is an inflammatory neurodegenerative disease caused by autoimmune attack against myelin [28]. So far, the understanding of the pathogenesis of MS is still very limited. Currently, some drugs, such as interferon β, glatirmer acetate, mitoxantrone, natalizumab and so on, have been used in MS patients [29, 30]. However, these treatments can not treat MS completely because of low efficiency or life-threatening side effects, it is very important to find out new candidate drugs for MS therapy.

It is now known that histamine takes part in several physiological and pathological processes, such as the allergic skin reactions, septic inflammation, etc [31]. The functions of histamine are mediated through four G-protein coupled receptors (H1R, H2R, H3R, H4R). So far, many GPCRs including histamine receptors are reported to play important roles in the pathogenesis of MS [32]. H1R and H4R stimulation leads to worsening of MS, whereas the signaling activation of H2R and H3R leads to amelioration of disease [12]. According to the reports from Teuscher et al., the deficiency of H3R could aggravate EAE symptom in mice [16]. Therefore, H3R may be a potential target to treat MS/EAE. To address the possibility of H3R as a target for MS treatment, EAE mouse model was induced and the therapy was performed with immethridine, a strong and highly selective histamine H3 receptor agonist. The results showed that immethridine-treated EAE mice had lower clinical scores and less pathological characteristics. These data strongly suggested that immethridine had the therapeutic effect on EAE. Furthermore, we also evaluated cytokine expression and T cell subgroups of splenocytes in EAE mice, the data indicated that lower level of IFN-γ and IL-17A and less Th1 and Th17 cells were detected in immethridine-treated EAE mice. However, H3R didn’t express on T cells and it has been confirmed by RT-PCR in current study (data not shown).

Dendritic cells (DCs) link innate immunity and adaptive immunity as professional antigen presenting cells (APCs) and play important roles in maintaining central and peripheral tolerance [33], the functional abnormal of DCs can lead to various autoimmune and neuroinflammatory diseases [34-36]. Interestingly, lower level of surface molecule expression was detected in immethridine-treated mice. To understand if immethridine can affect the functions of DCs and present the therapeutic effect on EAE, bone marrow-derived dendritic cells (BMDCs) were prepared and the biological functions of DCs were studied with or without immethridine. The results of cytokine expression showed that immethridine could inhibit the cytokine secretion such as IL-12, IL-6 and TGF-β, which were associated with the differentiation of Th1 and Th17. And the result has also been verified by cell differentiation assay. The results of cell differentiation suggested that the cell culture supernatant derived from immethridine-treated DCs could induce more Naïve CD4^+^ T cells to differentiate into Th1 and Th17, which played important roles in the pathogenesis of MS/EAE [37, 38]. The expression level of surface molecules such as CD40 and CD86 is closely related to the maturation of DCs. In the study, we found that immethridine could inhibit the expression of CD40 and CD86 both in immature or LPS-stimulated DCs, which meant that the maturation of DCs could be inhibited by the activation of H3R with immethridine. The ability of antigen presentation of DCs was assayed *in vitro* and the results indicated the treatment of immethridine reduced the function of antigen presentation and the inhibitory effect has also been detected in MLR assay. The maturation of DCs could be induced by LPS stimulation via activating NF-κB or MAPK signaling pathway [39, 40]. To understand what kind of signaling pathway was involved in the function of immethridine, the signaling pathway of NF-κB and MAPK was analyzed and the results indicated that immethridine could reduce the expression of TLR-4 and CD14, which are receptor or co-receptor of LPS, and down-regulate the phosphorylation of NF-κB p65 but not ERK1/2.

In summary, our data demonstrated that immethridine had therapeutic effect on EAE and could inhibit the function of DCs through activating H3R, such as cytokine production, surface molecule expression, T cell differentiation, and antigen-presenting ability. As the pathogenesis of MS or EAE remains unknown, effective treatments need to be developed, our results may provide a new therapeutic target for MS/EAE.

## MATERIALS AND METHODS

### Mice

Female C57BL/6 mice of 8-10 weeks old were purchased from Laboratory Animal Center of East China Normal University. Animal care and experimental procedures were carried out following the guidelines of the institutional Animal Ethics Committee of East China Normal University.

### EAE induction and treatment

EAE model was induced as previously described [41]. Briefly, Female mice at 8-10 wk of age were immunized subcutaneously in the flanks with 200 μg of MOG_35-55_ peptides (purity >98%, GL Biochem Ltd., Shanghai) which were emulsified in complete Freund’s adjuvant (CFA) containing 500 μg of Mycobacterium tuberculosis H37RA (Difco Laboratories, USA). Furthermore, 400 ng of pertussis toxin (PTX, List Biological Laboratories, USA) in 100 μl PBS-50 mM NaCl was intraperitoneally injected on the day of immunization and 24 h later. For the treatment of EAE, immethridine dihydrobromide (Sigma-Aldrich, USA) was administered at 15 mg/kg i.p. every other day after the observable sign of EAE occurred. Mice were monitored every day using the following arbitrary scale: 0, no symptom; 1, limp tail; 2, limp tail and wobbly gait; 3, bilateral hind limb paralysis; 4, complete hind limb and partial fore limb paralysis; 5, Death.

### Histopathological analysis

On day 21, animals were humanly euthanatized and their sera, spleen, brain hemisphere (without the cerebellum) and the spinal cord were isolated for subsequent experiments. Spinal cord samples were then fixed in 4% (w/v) paraformaldehyde for 48h. Paraffin-embedded sections were stained with hematoxylin-eosin (H&E) or luxol fast blue (LFB) to analyze inflammation or demyelination, respectively.

### MOG-specific T cell proliferation assay

For ex vivo assay, splenocytes were isolated from immethridine-treated or control EAE mice and cultivated in triplicates in complete RPMI1640 media (with 10% FBS) at 2×10^5^ per well in 96-well plates in presence or absence of the MOG_35-55_ peptide (20 μg/ml), all cultures were maintained at 37 °C in 5% CO_2_ for 72 h. T cell proliferation was measured using a MTT Cell Proliferation Assay Kit (Beyotime, China). Briefly, 10 μl of MTT (methylthiazolyldiphenyl-tetrazolium bromide) solution (5 mg/ml) was added to each well and incubated for further 4 h. 100 μl of formazan solvent was added to each well and incubated until all formazan was dissolved and the absorbance was evaluated by a microplate reader at 570 nm. The results were expressed as a stimulation index (SI, SI=OD570 of experimental cells / OD570 of negative control), which indicates significant proliferation if SI ≥2.

### Analysis of CD4^+^T cell subgroups and DCs in the spleen of EAE mice

Splenocytes from immethridine-treated or control EAE mice were collected, washed, and incubated in Red Blood Cell Lysis Buffer at room temperature for 5 min, single cell suspensions were prepared. CD4^+^ T cell subgroups were detected by intracellular cytokine staining (ICCS) as previously described. Briefly, the cells were washed and restimulated with 10 ng/ml PMA (Sigma) plus 1 μg/ml ionomycin (Calbiochem, San Diego, CA) for 5 h. Brefeldin A (Sigma, 10 μg/ml) was added during the last 2 h of culture. After the surface staining with FITC-labeled anti-CD4 mAb (BD Biosceince), cells were fixed, permeabilized, stained with PE-labeled anti-IFN-γ, anti-IL-17A mAb or anti-Foxp3 mAb (BD Biosciences), and analyzed on a flow cytometer (Becton Dickinson, USA) using CellQuest software (BD Biosciences). The expression of surface molecules were detected with FITC-labeled anti-CD11c mAb (BD Biosciences) and PE-labeled anti-CD40, anti-CD86 or anti-MHCII mAb (BD Biosciences).

### Preparation of bone marrow-derived dendritic cells

Bone marrow-derived dendritic cells (BMDCs) were prepared as described previously [42]. Briefly, femurs and tibias of 6-8 week-old mice were removed and isolated from the surrounding muscle tissue using sterile instruments. The bones were washed three times with phosphate-buffered saline (1×PBS), and then the bone marrow was flushed with RPMI-1640 media (HyClone, USA) using a 1-ml syringe. After the red blood cells were lysed with Red Blood Cell Lysis Buffer, whole bone marrow cells were counted. Cells obtained from femurs and tibias were cultured at a final concentration of 1×10^6^ cells/ml in six-well plates in the culture media containing RPMI-1640, 10% fetal bovine serum (FBS) (HyClone, USA), 10 ng/ml recombinant murine GM-CSF (Peprotech, USA), 1 ng/ml IL-4 (Peprotech, USA), 1 U/ml penicillin and 1 μg/ml streptomycin, at 37 °C, 5% CO_2_ for 6 days. Then the media were replaced with fresh, pre-warmed complete media at day 2 and day 4 to remove non-adherent cells. On day 6, maturation of immature DCs was induced by the addition of 100 ng/mL LPS.

To analyze the purity of DCs, the cells were stained with FITC-labeled anti-CD11c mAb (BD Biosciences, San Jose, CA) and analyzed by flowcytometry. The purity of DCs was up to 85-90%.

### RT-PCR or quantitative real-time PCR analysis of gene expression

Total RNAs were extracted using Trizol Reagent (Takara, Japan) and the reverse transcription was performed using Prime-ScriptTM RT Reagent Kit according to the manufacturer’s instructions (Takara, Japan). The mRNA expression was determined by RT-PCR or quantitative real-time PCR (Q-PCR). PCR products were analyzed on a 1% agarose gel stained with ethidium bromide. Q-PCR was conducted in the LightCycler Quantitative PCR Apparatus (Illumina, USA) using the SYBR Green Master Mix (Toyobo, Japan) and the results were presented as fold increases relative to the expression of house keeping GAPDH. The following primer pairs were used: IL-6, forward, 5’- TTCTTGGGACTGATGCTG-3’, and reverse, 5’-CTGGCTTTGTCTTTCTTGTT--3’; TGF-β, forward, 5’-CCGCAACAACGCCATCT-3’, and reverse, 5’-GCCCTGTATTCCGTCTCCTT-3’; IL-12 P40, forward, 5’-CCAAGAACTTGCAGATGAAGC-3’, and reverse, 5’-TTCCTTTCCAACGTTGCATC-3’; TNF-α, forward, 5’-TCCCTTTCACTCACTGGC-3’, and reverse, 5’-ACTTGGTGGTTTGCTACG-3’; IFN-γ, forward, 5’-CACTGCATCTTGGCTTTGCA-3’, and reverse, 5’-GCTGAT GGCCTGATTGTCTTTC-3’; IL-17A, forward, 5’-CTCAAAGCTCAGCGTGTCCAA-3’, and reverse, 5’-TCATGTGGTGGTCCAGCTTTC-3’; TLR4, forward, 5’-ATGGCATGGCTTACACCACC-3’, and reverse, 5’-GAGGCCAATTTTGTCTCCACA-3’; CD14, forward, 5’-CTCTGTCCTTAAAGCGGCTTAC-3’, and reverse, 5’-GTTGCGGAGGTTCAAGATGTT-3’; GAPDH, forward, 5’-GGGCATCTTGGGCTACACT-3’, and reverse, 5’-GCCGAGTTGGGATAGGG-3’.

### ELISA analysis of cytokine production

DCs were stimulated with immethridine (1 μM) and LPS (100 ng/ml) for 72h, the supernatants were collected and stored at -80 °C until the cytokine measurements. The assays for IL-6, TGF-β and IL-12 were performed with ELISA kits from eBioscience according to the manufacturers’ protocol.

### FACS analysis of surface molecule expression

Immature DCs exposed to combinations of immethridine (1 μM) and/or LPS (100 ng/ml) for 12 h were stained with a FITC-labeled anti-CD11c and PE-labeled anti-CD40 or anti-CD86 (BD Biosciences, San Jose, CA). FACS analysis was performed on a flow cytometer (Becton Dickinson, USA) using CellQuest software (BD Biosciences). Results are expressed as mean fluorescent density (MFI).

### T cell differentiation induced by DCs

DCs were treated with immethridine (1 μM) and LPS (100 ng/ml) for 24h. Naïve CD4^+^ T cells were prepared by Mouse CD4 Negative Selection Kit (Stemcell Technology, Canada) from spleens of C57BL/6 mice and co-cultured with immethridine-treated or untreated DCs at a ratio of 10:1 for 72h at the present of 0.5 μg/ml anti-CD3 mAb and 5 μg/ml anti-CD28 mAb (BD Biosciences). The percentage of Th1 and Th17 cells was analyzed by intracellular staining with FITC-labeled anti-IFN-γ or FITC-labeled anti-IL-17A (BD Biosciences, San Jose, CA).

### Antigen-presentation assay

DCs were incubated with immethridine and MOG_35-55_ (20 μg/ml) for 4h. After washed with PBS, DCs were stimulated with LPS (100 ng/ml) for 12h. 1×10^6^ of CD4^+^ T cells, isolated from splenocytes of EAE mice with Mouse CD4 Negative Selection Kit (Stemcell Technology, Canada), were co-cultured with 1×10^5^ of MOG_35-55_ peptide-pulsed DCs at a ratio of 10:1 ratio in 96-well round bottom plates for 72h. Finally, cells were analyzed by MTT Cell Proliferation Assay Kit according to the manufacturer’s instructions.

### Mixed lymphocyte reaction (MLR)

T cells were purified from splenocytes of BALB/c mouse using T cell Enrichment Columns (R&D Systems, Minneapolis, MN) and were used as responders (1×10^5^/well). The bone marrow-derived DCs from C57BL/6 mice were prepared as before. The harvested DCs were treated with immethridine (1 μM) or PBS for 4 h in a humidified atmosphere with 5% CO_2_ at 37 °C. Then DCs were treated with 30 μg/ml Mitomycin C (Sigma, USA) for 45 min. After being washed 3 times by RPMI 1640, they were used as stimulators. For the induction of allogeneic MLR, purified T cells were co-cultured with immethridine-treated or untreated allogeneic DCs at the DC/T cell ratio of 1:5, 1:10 and 1:20 in triplicate in flat-bottom 96-well microplates. Negative control received only T cells and media. The plates were incubated at 37 °C in a humid atmosphere with 5% CO_2_ for 5 days and the result was measured by MTT Cell Proliferation Kit (Beyotime, China) according to the manufacturer’s instructions.

### Western blot analysis of signaling pathway

DCs were treated with 100 ng/ml LPS and 1 μM immethridine for 12h and the whole-cell extracts were prepared by RIPA buffer supplemented with different kinds of proteinase inhibitors. Proteins were loaded onto a 8% SDS-polyacrylamide gel and subjected to electrophoresis. The separated proteins were electro-transferred onto a nitrocellulose membrane using a Mini Trans-Blot apparatus (Bio-Rad). The membrane was then blocked with skimmed milk and probed with specific antibodies against p65 (pSer536-p65), ERK1/2 (pThr202/Tyr204-ERK1/2) (Cell Signaling Technology, USA).

### Statistical analysis

Student’s t test was used to analyze the differences between different groups. Data were presented as mean ± standard deviation (SD). Difference was considered statistically significant when p < 0.05.

## Abbreviations

ADHD: attention-deficit hyperactivity disorder
Ag: antigens
APC: antigen presenting cells
BMDC: bone marrow-derived dendritic cells
CNS: central nervous system
DC: dendritic cell
EAE: experimental autoimmune encephalomyelitis
GPCR: G protein-coupled receptor
H_3_R: histamine H_3_-receptor
LPS: lipopolysaccharide
MLR: mixed lymphocyte reaction
MS: multiple sclerosis
Q-PCR: quantitative real-time PCR
